# Saliva from Obese Individuals Suppresses the Release of Aroma Compounds from Wine

**DOI:** 10.1371/journal.pone.0085611

**Published:** 2014-01-22

**Authors:** Paola Piombino, Alessandro Genovese, Silvia Esposito, Luigi Moio, Pier Paolo Cutolo, Angela Chambery, Valeria Severino, Elisabetta Moneta, Daniel P. Smith, Sarah M. Owens, Jack A. Gilbert, Danilo Ercolini

**Affiliations:** 1 Department of Agricultural Sciences, University of Naples Federico II, Portici, Italy; 2 General and Laparoscopic Surgery Unit, S., Giovanni Bosco Hospital, Naples, Italy; 3 Department of Environmental, Biological and Pharmaceutical Sciences and Technologies, Second University of Naples, Caserta, Italy; 4 IRCCS, Multimedica, Milano, Italy; 5 Research Center on Food and Nutrition, Agricultural Research Council, Rome, Italy; 6 Argonne National Laboratory, Argonne, Illinois, United States of America; 7 Computation Institute, University of Chicago, Chicago, Illinois, United States of America; 8 Department of Ecology and Evolution, University of Chicago, Chicago, Illinois, United States of America; Wageningen University, The Netherlands

## Abstract

**Background:**

Recent evidence suggests that a lower extent of the retronasal aroma release correspond to a higher amount of *ad libitum* food intake. This has been regarded as one of the bases of behavioral choices towards food consumption in obese people. In this pilot study we investigated the hypothesis that saliva from obese individuals could be responsible for an alteration of the retro-nasal aroma release. We tested this hypothesis *in vitro*, by comparing the release of volatiles from a liquid food matrix (wine) after its interaction with saliva from 28 obese (O) and 28 normal-weight (N) individuals.

**Methods and Findings:**

Amplicon sequencing of the 16S rRNA V4 region indicated that *Firmicutes* and *Actinobacteria* were more abundant in O, while *Proteobacteria* and *Fusobacteria* dominated in N. *Streptococcaceae* were significantly more abundant in the O subjects and constituted 34% and 19% on average of the saliva microbiota of O and N subjects, respectively. The Total Antioxidant Capacity was higher in O *vs* N saliva samples. A model mouth system was used to test whether the in-mouth wine aroma release differs after the interaction with O or N saliva. In O samples, a 18% to 60% significant decrease in the mean concentration of wine volatiles was detected as a result of interaction with saliva, compared with N. This suppression was linked to biochemical differences in O and N saliva composition, which include protein content.

**Conclusion:**

Microbiological and biochemical differences were found in O *vs* N saliva samples. An impaired retronasal aroma release from white wine was detected *in vitro* and linked to compositional differences between saliva from obese and normal-weight subjects. Additional *in vivo* investigations on diverse food matrices could contribute to understanding whether a lower olfactory stimulation due to saliva composition can be a co-factor in the development/maintenance of obesity.

## Introduction

Determining the factors that drive food choice is essential to aid in the promotion of healthy eating habits. An individual's food choice can depend on several factors, including sensory perception, which affects food/beverage preference and liking, and also satiation and subsequent eating/drinking inhibition [1]–[3]. Inter-individual variability in sensory perception is large and genetic or cognitive factors have been studied to explain such variability. Nevertheless, during eating/drinking, food is mixed with saliva and the products of the food-saliva interaction are perceived rather than the food itself. Therefore, together with its main functions (e.g. speech, maintaining oral and general health, and food processing), saliva has a role in the appreciation and acceptance of food/beverage [4]. During eating/drinking, all kinds of oral sensation (taste, viscosity, astringency, etc.) are modulated by saliva [5]. In addition, the retronasal olfactory perception, arising when the odorants interact with odour receptors by migrating from the mouth to the nasal cavity via the nasopharynx, is significantly affected by the interaction with saliva. Previous results have demonstrated the role of saliva on the retronasal aroma perception of wine [6] which, combined with similar findings on other food matrices [7], led to the development of the hypothesis that saliva-derived interactions with food/beverage have a significant role in food preference, perception and therefore personal diet selection. We question if diseases resulting in an alteration of saliva composition modify the food/beverage sensory perception of an individual and consequently their preferences, choices and habits. Can this alteration have an impact on Body Mass Index (BMI)?

The World Health Organization (WHO) defined obesity as “The global health emergency”. Worldwide, around 250 million people are obese, and the WHO has estimated that in 2025, 300 million people will be obese [8]. The direct costs of obesity are now estimated to be around 7% of total health care costs in the United States and around 1%–5% in Europe [8]. This has led to an increase in global research regarding why some people eat more than necessary, and specifically what factors and mechanisms lead to maintenance of active appetite signals. The degree to which individuals gain pleasure from eating has been shown to have a genetic origin [9], as well as originating from sensory sensitivity [10], [11]. Findings are controversial, but it seems that metabolic disorders can perturb normal olfactory physiology and function [12].

Some non-metabolic factors can also affect the olfactory perception during eating. It is known that the food/drink characteristics and other interpersonal differences that are uncontrolled by a person (nasal and mouth anatomy, oral processing habits and saliva), are important for the efficiency of retronasal aroma release with a consequent effect on satiation [2], [13]–[19].

In light of these evidences, we tested the hypothesis that in obese individuals, saliva could be responsible for an alteration of the retro-nasal aroma volatilization. This could have an impact on the delay of satiation due to a lower olfactory stimulation although only *in vivo* studies could support this hypothesis. A possible implication of saliva on the amount of food intake in obese people is supported by previous research, which shows that slower habituation of salivary responses to food stimuli is related to greater energy intake, and that obese individuals habituate slower than normal-weight. These findings suggest that decline in sensory responding to food occurs more slowly in obese individuals, possibly contributing to delayed satiation and greater food intake [20]–[23]. In hour hypothesis this lower responsiveness could be affected by a minor extent of the retronasal aroma release due to saliva composition.

In this study, the microbiological and biochemical inter-individual variability in saliva samples from 28 obese and 28 normal-weight individuals, was determined by 16S rRNA V4 amplicon sequencing and proteomics approaches. These saliva analyses were performed by taking into account that macromolecules from both bacterial cell surface and from protein content of saliva could interact with aroma molecules of food. We also monitored some biochemical parameters linked to oxidative phenomena. The interest towards these parameters is not only for the purpose of characterization, but also takes account that an altered oxidative status could affect certain sensory-active substances sensitive to oxidation, such as volatiles. Afterwards, a model mouth system was used (Retronasal Aroma Simulator device), to test whether the extent of the retro-nasal aroma release from a real liquid food matrix (white wine) differs after the interaction with saliva from obese or normal-weight subjects. In this pilot study wine was used just as a model to mime saliva-volatiles interaction and not for its nutritional properties or for its relevance in obesity. Our results show that the biochemical and microbial composition of saliva differed between obese and normal-weight subjects. Additionally, our in vitro experiments showed that saliva from obese individuals interacted with aroma molecules leading to a decrease in the extent of oral aroma release. This suggests that when drinking wine, obese individuals may perceive less and different aromas than normal-weight individuals. To validate these results, full clinical trials on several food/beverage matrices are needed to better understand if an altered sensory perception due to saliva composition could be a driver of the feeding habits of obese individuals, and whether this could be a co-factor in the occurrence/maintenance of the disease.

## Results

### Microbiota variability between obese (O) and normal-weight (N) saliva

**Figure 1:**
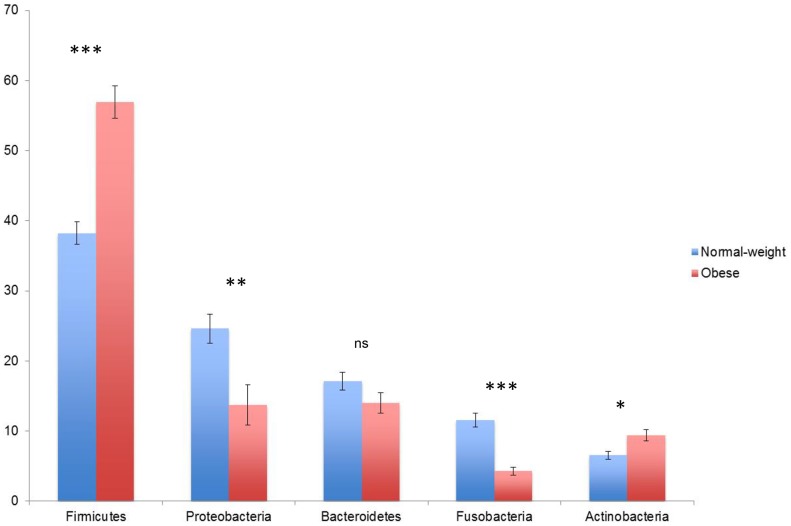
Average abundance of phyla in salivary samples of N and O subjects. Major bacterial phyla in saliva of normal-weight (N) and obese (O) subjects (*P<0.01, **P<0.001, ***P<0.0001, ns: not significant). Bars indicate standard error.

**Figure 2:**
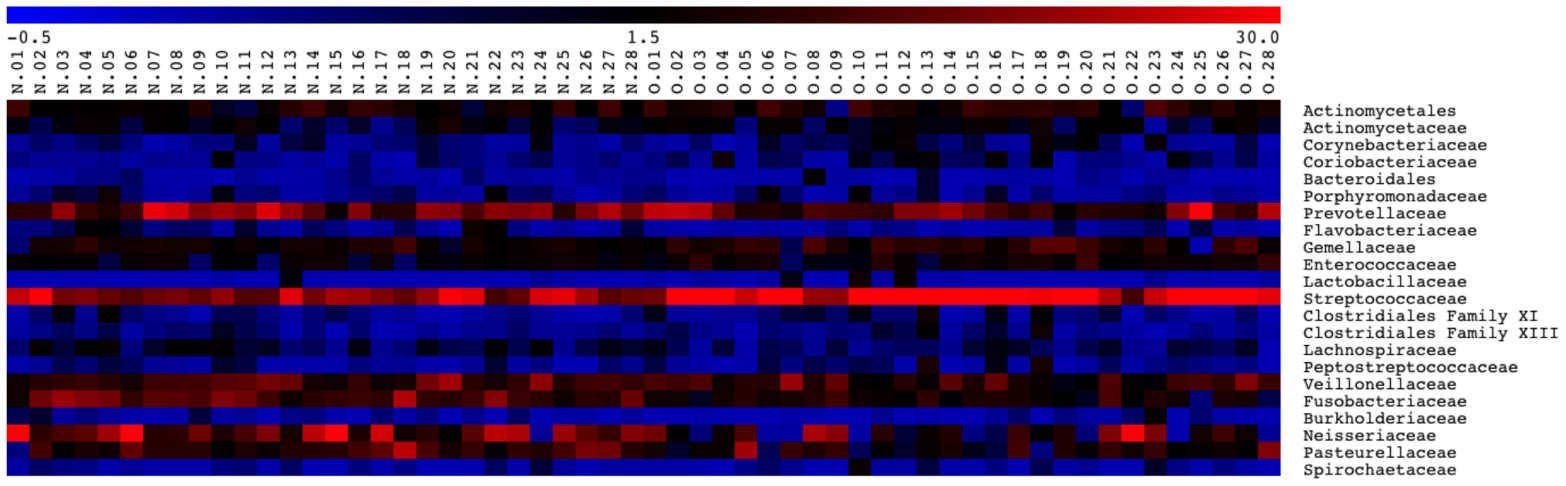
Abundance of bacterial families in salivary samples of N and O subjects. Microbial community structure and relative abundance of principal OTUs in saliva of normal-weight (N) and obese (O) subjects. Legend and scale shown in the upper part of the figure represent colors in the heat map associated with the relative percentage of each OTU within the samples.

**Figure 3:**
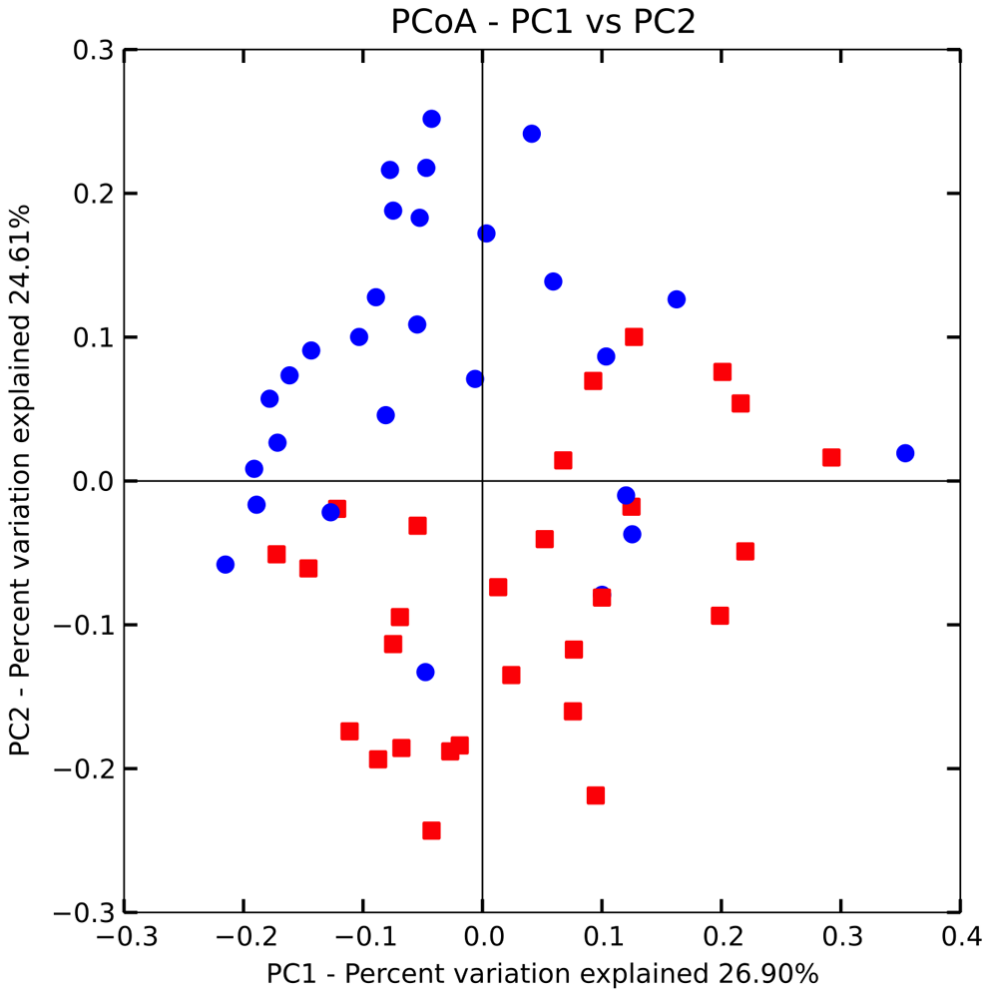
Principal Coordinates Analysis of weighted UniFrac distances for 16S rRNA gene sequence data. The distribution shows distances within and across normal-weight (N, red ▪) and obese (O, blu •) subjects populations on the basis of the microbiota.

A total of 56 saliva samples from 28 O and 28 N donors (Table 1) were analyzed with 4000 randomly-selected 16S rRNA sequence reads from each sample. A total of 3219 non-singleton Operational Taxonomic Units (OTUs) were identified among the 224,000 reads. Twelve phyla were identified, but only 5 dominated. With the exception of *Bacteroidetes*, the abundance of the phyla differed significantly (P<0.01) according to the subject type (obese: O *vs* normal: N). The *Firmicutes* and *Actinobacteria* were more abundant in obese samples, whereas *Proteobacteria* and *Fusobacteria* dominated in normal-weight subjects (Figure 1). With deeper taxonomic assignment *Streptococcaceae*, *Prevotellaceae*, *Fusobacteriaceae*, *Veillonellaceae*, *Neisseriaceae* and *Pasteurellaceae* were the most abundant OTUs in the saliva samples analyzed (Figure 2). The sample type and the obesity class significantly influenced the composition of the bacterial community as measured using ADONIS, ANOSIM, and MRPP methods (P<0.001). In particular, *Streptococcaceae*, *Gemellaceae* and *Enterococcaceae* were more abundant in obese, while *Fusobacteriaceae*, *Veillonellaceae*, *Prevotellaceae*, *Flavobacteriaceae* and *Lachnospiraceae* dominated in normal-weight subjects (Figure 2). *Streptococcaceae* constituted 34% of the saliva microbiota of O subjects compared to only 19% in N subjects. By contrast, *Fusobacteriaceae* were on average 19% of the population in N and only 4% in O subjects. The similarity of microbial community structures both within and between the two categories (O *vs* N) was evaluated by generating a weighted Unifrac distance matrix. The PCoA shows how the 2 categories of individuals clustered separately on the basis of the microbiota (Figure 3). Statistical correlation between single OTUs within the microbial community structure and all the other variables (i.e. BMI, occurrence of specific differentially expressed proteins, quantity of each compound released after saliva interaction with wine, etc.) identified statistically significant correlations in the following cases: the presence of *Streptococcaceae* (r = 0.55), *Gemellaceae* (r = 0.47) and *Enterococcaceae* (r = 0.40) were positively correlated to BMI; the occurrence of *Fusobacteriaceae* (r = −0.49) and *Prevotellaceae* (r = −0.48) were negatively correlated to BMI (P<0.001). However, no correlation was found between presence or relative abundance of microbial groups and volatile compounds released from wine.

**Table 1:** Description of saliva donors.

	O		N
Sex	male (n = 28)		male (n = 28)
Age (years)	21–29 (n = 10)		20–27 (n = 11)
	30–36 (n = 4)		30–39 (n = 7)
	42–47 (n = 7)		40–48 (n = 6)
	52–58 (n = 5)		51–52 (n = 3)
	64–68 (n = 2)		60 (n = 1)
Normal weight			
(18.5≤BMI*≤24.9)		n = 28	
Obese class I			
(30≤BMI≤34.9)	n = 8		
Obese class II			
(35≤BMI≤39.9)	n = 4		
Obese class III			
(BMI≥40)	n = 16		
Smokers	n = 10		n = 9

O: obese; N: normal-weight.

n: number of donors.

* Body Mass Index (BMI, Kg/m2) categories are defined according to the World Health Organization guidelines (http://www.who.int/bmi).

### Biochemical variability between O and N saliva

**Figure 4:**
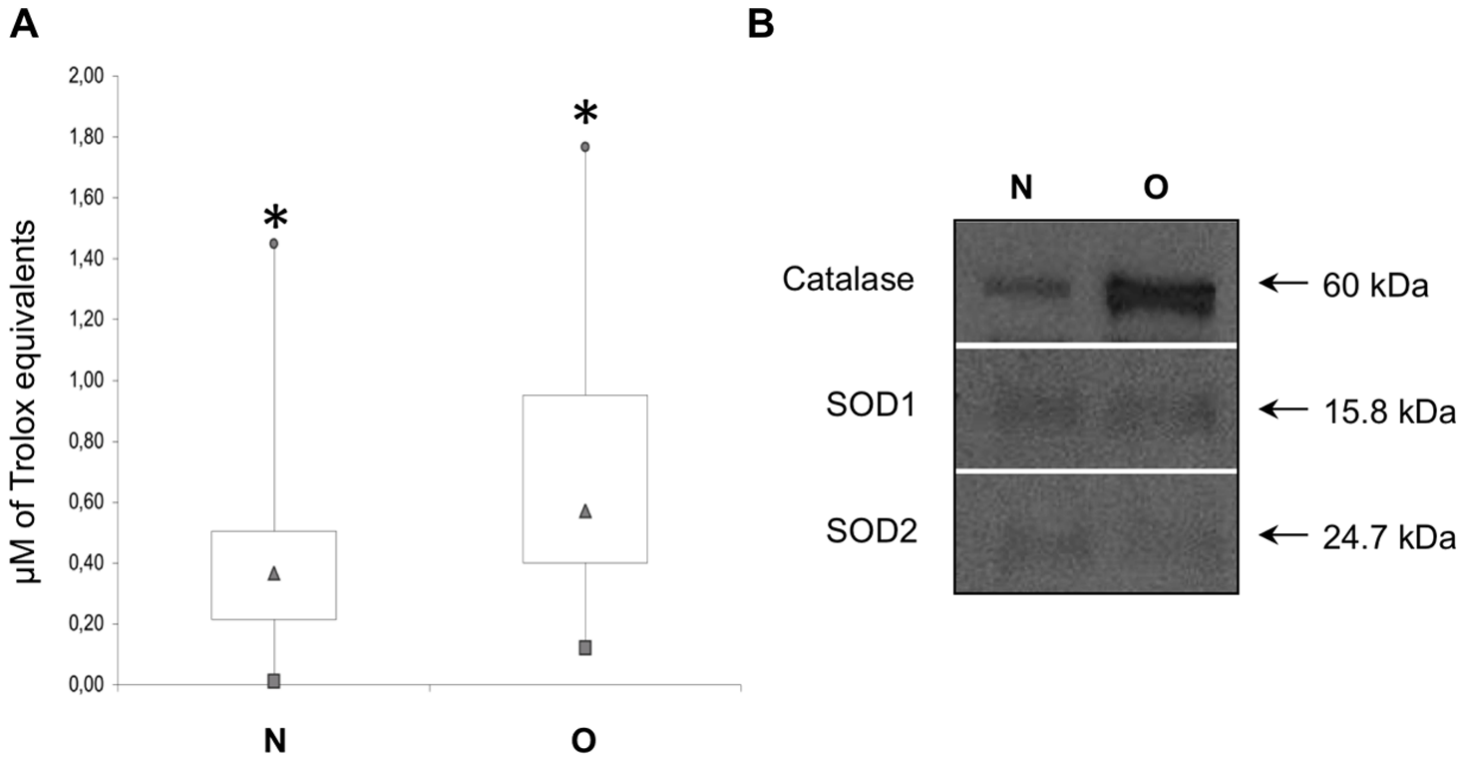
Biochemical variability in salivary samples of N and O subjects. Box plot representation of Total Antioxidant Capacity of normal-weight (N) and obese (O) salivary samples. Box outline represents lower and upper quartiles, squares and circles correspond to the smallest and largest observations, the triangle is the median value; *P≤0.01 (A). Western blotting analysis of catalase, SOD1 and SOD2 (B).

The Total Antioxidant Capacity (TAC) was significantly higher (P<0.01) in the O compared to N samples with median values of 0.57 and 0.36 µM of Trolox Equivalents (TE), respectively (Figure 4A). In addition, a higher inter-individual variability was observed for the obese subjects with interquartile ranges of 0.21–0.49 (N) *vs.* 0.40–0.95 (O). Accordingly, higher expression levels of the antioxidant enzyme catalase were revealed in the O samples (Figure 4B). No significant differences were observed for the copper-zinc (SOD1) and the manganese (SOD2) superoxide dismutases that were minimally detected in both samples. Inter-individual variability was also observed for total protein concentration of the O and N salivary samples with mean values of 1.2 mg/mL and 1.5 mg/mL, respectively, in agreement with the physiologic protein content reported for human whole saliva (i.e. 0.5–3 mg/mL) [24].

Protein analysis identified a major band with molecular weight of approximately 58 kDa, later identified as salivary alpha-amylase; it was detected in SDS-PAGE protein profiles of all samples. Several other protein bands were also visualized in the regions with molecular masses in the range of 14–17 kDa. Protein profiles were analyzed and selected proteins showing the major variability between N and O samples were identified by MALDI-TOF MS (Table 2). Several proteins of glandular origin including alpha-amylase, cystatins, lactotransferrin, lysozyme, as well as proteins known to have originated from immune cells (cathepsin G), were identified. Additionally, serum derived albumin and the calcium/phospholipid-binding protein annexin A2, were also identified.

**Table 2:** Proteins identified by MALDI-TOF MS. Protein theoretical relative mass (Mr) and isoelectric point (pI) are reported along with the coverage percentages, the number of matching peptides and the Mascot identification score.

Accession #	Description	Mr/pI	Coverage (%)	Peptides #	Mascot Score
P63261	Actin cytoplasmic 2	42108/5,31	55	22	64
P04745	Alpha-amylase 1 salivary	58415/6,47	61	34	91
P07355	Annexin A2	38808/7,57	74	33	103
P08311	Cathepsin G	29161/11,19	57	18	101
P01036	Cystatin-S	16489/4,95	80	15	97
P01037	Cystatin-SN	16605/6,73	73	12	72
Q8TCD2	Lactotransferrin	80014/8,50	59	48	156
P61626	Lysozyme C	16982/9,38	99	19	50
P01833	Polymeric Ig receptor	84429/5,58	52	37	72
Q9P157	Serum albumin	71317/5,92	65	40	133
Q08188	Transglutaminase E	76926/5,62	56	38	62
Q4V348	Zinc finger protein 658B	97349/8,90	53	60	81

### In-mouth aroma release variability between O and N saliva

Twenty-two aroma compounds were detected in the dynamic headspace of the model mouth system after the interaction of white wine with O or N saliva. In Table 3 they were listed and grouped according to their chemical class: 10 esters, 4 alcohols, 4 norisoprenoids, 2 aldehydes and 1 acid. For each compound the mean concentration calculated on the 28 O and on the 28 N saliva samples, was reported. On average, the extent of aroma release after interaction with saliva was different between obese and normal weight subjects. Many of the differences between the mean values were statistically significant (Table 3). The percentage change in the concentration of wine aroma compounds released in the model mouth headspace after interaction with the two different saliva types was also calculated. We found that, compared to N, the wine interaction with O saliva samples resulted in attenuation of all white wine aromas that may reach the olfactory receptors in the nasal cavity by the retronasal pathway (minimum significant loss: 18%; maximum significant loss: 60%). Specifically, a significant reduction from 49% (ethyl acetate) to 18% (2-phenylethyl acetate) in esters concentration was detected. Saliva from obese resulted in an even more extensive impact on alcohols and on the only acid detected: floral 2-phenylethanol was attenuated by 60% and hexanoic acid by 54%. Among the aldehydes and norisoprenoids, the negative impact of obese saliva was more extensive on the aldehydes, with the two almond-like odorants furfural and benzaldehyde attenuated by 41 and 21%, respectively (Table 3).

**Table 3:** Volatile compounds detected in the dynamic headspace of the white wine in the presence of saliva from obese and normal-weight subjects.

Compounds	Mean concentrations (mg/L)"						Wilcoxon-Mann-Whitney		O *vs.* N	Odor descriptors#
		non-parametric test		(%) §	
	O	±	SD	N	±	SD	U	P value		
**Esters**										
ethyl octanoate	8,684	±	3,591	10,391	±	4,827	145	ns	−16	fruity wine waxy sweet apricot banana brandy pear
ethyl acetate	6,656	±	4,817	13,116	±	10,508	96	*	−49	ethereal fruity sweet weedy green
ethyl hexanoate	3,097	±	1,089	3,645	±	1,546	136	ns	−15	sweet fruity pineapple waxy green banana
ethyl decanoate	1,867	±	0,936	2,270	±	1,261	149	ns	−18	sweet waxy fruity apple grape oily brandy
3-methylbutyl acetate	1,006	±	0,444	1,233	±	0,494	132	ns	−18	fruity, banana
ethyl-9-decenoate	0,298	±	0,113	0,347	±	0,156	154	ns	−14	fruity fatty
ethyl butanoate	0,226	±	0,143	0,307	±	0,137	122	ns	−26	fruity juicy fruit pineapple cognac
ethyl lactate	0,209	±	0,129	0,363	±	0,276	107	*	−43	sharp tart fruity buttery butterscotch
ethyl succinate	0,188	±	0,104	0,317	±	0,222	96	*	−41	mild fruity cooked apple ylang
2-phenylethyl acetate	0,012	±	0,003	0,015	±	0,006	113	*	−18	floral rose sweet honey fruity tropical
**Alcohols**										
ethanol	163,303	±	138,699	324,699	±	288,916	107	*	−50	strong alcoholic ethereal medical
3-methyl-1-butanol	6,087	±	3,033	7,437	±	3,986	138	ns	−18	fusel oil alcoholic whiskey fruity banana
2-phenylethanol	0,236	±	0,163	0,589	±	0,494	74	**	−60	floral rose dried rose flower rose water
1-hexanol	0,128	±	0,027	0,145	±	0,034	119	ns	−11	ethereal fusel oil fruity alcoholic sweet green
benzyl alcohol	0,004	±	0,002	0,009	±	0,002	63	***	−51	floral rose phenolic balsamic
**Norisoprenoids**										
vitispirane I	0,052	±	0,013	0,056	±	0,025	164	ns	−8	floral fruity earthy woody
vitispirane II	0,048	±	0,013	0,052	±	0,024	176	ns	−7	floral fruity earthy woody
TDN	0,039	±	0,017	0,045	±	0,024	158	ns	−14	kerosene
beta-damascenone	0,002	±	0,000	0,002	±	0,000	141	ns	−12	natural sweet fruity rose plum grape raspberry sugar
**Aldehydes**										
furfural	0,149	±	0,068	0,253	±	0,158	98	*	−41	sweet woody almond fragrant baked bread
benzaldehyde	0,005	±	0,001	0,006	±	0,001	63	***	−21	strong sharp sweet bitter almond cherry
**Acid**										
hexanoic acid	0,098	±	0,056	0,212	±	0,155	70	**	−54	sour fatty sweat cheese

O: obese; N: normal-weight.

" Mean values ± standard deviation of 28 saliva samples.

§ Percentage change in the concentrations of aroma compounds after interaction with saliva from obese (O) with respect to saliva from normal weigh (N) subjects.

# Odor descriptors were from The Good Scent Company (http://www.thegoodscentscompany.com/search.html) online programme.

Significantly different values: * P<0.05, ** P<0.01, ***P<0.001.

ns: not significant.

A Partial Least Square Discriminate Analysis (PLS-DA) was performed. Data collected on selected volatiles (only those reported as significantly different in Table 3) and biochemical parameters (differently expressed proteins, total proteins and TAC) constituted the independent X-block of variables, while saliva samples (O and N) represented the dependent Y-category.

**Figure 5:**
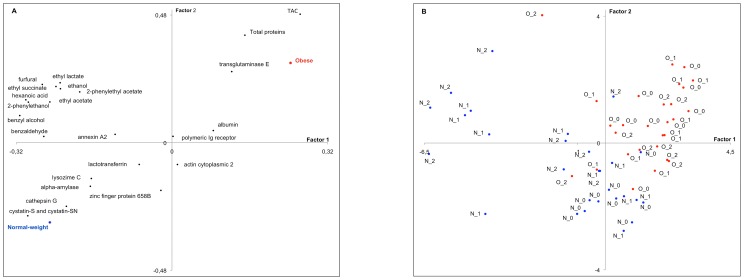
PLS-DA calibration modelling. Weighted loading plot (A) and scatter plot of scores (B) for X-variables, and their inner relationships with O (red •) and N (blu •) for the first two latent variables (43% of the total variance in the X-block; 56% of the total variance in the Y-block).

A significant four-factor PLS-DA model, explaining 53% and 69% of the data variance in the X and Y blocks, respectively, was obtained. Figure 5A shows the weighted loading plots for X-variables, and their inner relationships with O and N class for the first two latent variables (43% of the total variance in the X-block; 56% of the total variance in the Y-block). The scatter plot of scores of the first two components is also reported in Figure 5B. The latter shows a good distinction of the two saliva types (O and N) along the F1, with the N essentially divided into two groups. The corresponding weighted loading plot (Figure 5A) established the relative importance of each volatile component and biochemical parameter to the saliva samples. The results revealed that only some biochemical parameters (TAC, total proteins, transglutaminase E and albumin) are located in the higher right quadrant as well as O class. Along F2, most of the remaining proteins are on the negative side together with N class and with volatiles placed in the F2 positive side.

Weighted regression coefficients (BWs) provide information about the importance of the X-variables in explaining differences in O and N saliva: the higher the coefficient, the more correlated is the related X-variable to that Y. The selected volatile compounds and biochemical parameters with high significant BWs from PLS-DA calibration are summarized in Table 4, together with some additional information given by the loading factors 3 and 4 (plots not shown). The most relevant X-variables in terms of explained variance after 4 factors are: TAC, total proteins, albumin, lactotransferrin, transglutaminase E and 2-phenylethyl acetate for O samples; cystatin-S and cystatin-SN, alpha-amylase, benzaldehyde, cathepsin G, benzyl alcohol, ethyl acetate, hexanoic acid, 2-phenyethanol and furfural in the case of N samples.

**Table 4:** Relevance of the PLS-DA calibration modeling: explained variances and high positive weighted regression coefficients (BWs).

Number of factors	Explained variances (%)		Variables with high positive BW after 4 factors	
	X	Y	O	N
2	43	56	TAC	cystatin-S and cystatin-SN
			total proteins	alpha-amylase
4	53	69	albumin	benzaldehyde
			transglutaminase E	cathepsin G
			lactotransferrin	benzyl alcohol
			2-phenylethyl acetate	ethyl acetate
				hexanoic acid
				2-phenyethanol
				furfural

## Discussion

Different pathologies can provoke and cause biochemical and microbiological changes in saliva [25], [26]. However, rather surprisingly, saliva has been poorly investigated in relation to obesity. The microbiota of saliva has been linked to obesity [27], and an association between obesity and bacterial cellular abundance in oral subgingival biofilms was recently found in adolescents [28]. As observed in the latter study, we found an increased abundance of *Firmicutes* and *Actinobacteria* in obese subjects compared to those of normal-weight. Additionally, and similar to our current results, Zeigler and colleagues found that *Streptococcaceae* was significantly more abundant in obese, compared to normal-weight adolescents; however, unlike our study, they observed that *Fusobacteriaceae* occurred at higher percentages in obese subjects. Goodson and coworkers [27] observed *Selenomonas noxia* (*Veillonellaceae*) in saliva of obese women, while in this study (which only targeted taxa at the family level, and only involved male subjects) the *Veillonellaceae* were more abundant in normal-weight individuals.

It has been demonstrated that gut and oral microbiota can have common structures [29]. Indeed, a considerable amount of bacterial cells are swallowed daily with saliva [30]; the microbiota of saliva could therefore affect the gut microbiota composition although the resistance of such bacteria to gastrointestinal conditions has yet to be proven. In addition, diet, substrates and microbial competition can play a role in the possibility that salivary microbiota can establish in the gut. In the current study, *Firmicutes* was observed to have a significantly higher abundance in the saliva of obese subjects, which according to the literature may have a causative role in the development of obesity. In fact, the occurrence of specific bacteria such as *Firmicutes* in the gut can promote absorption of monosacchrides and play a role in the development of obesity [31], [32].

On the basis of our results, it was not possible to detect obesity-associated OTUs. It would be interesting to understand whether within the families that were found to be more associated with obese saliva (*Streptococcaceae*, *Gemellaceae* and *Enterococcaceae*) there is some particularly abundant microbial species that can be correlated to obesity. However, deeper taxonomic assignment would only be reliable with longer 16S rRNA reads, or by targeting alternative taxonomic marker genes. It is axiomatic that bacteria in saliva are responsible for metabolic activities leading to the release of aromatic compounds while chewing, although the degree to which this affects the retronasal olfactory response is unknown. Yet, physicochemical interactions between microbes and food can take place in the mouth and have an effect on the adsorption of volatiles from food matrices. Accordingly, microrganisms have been shown to hamper the release of volatile compounds from food due to the adsorption of odor molecules on cell walls [33]–[35]. However, our results indicated no correlation between the observed microbial populations and the volatile molecules detected after the contact between wine and saliva, suggesting either a negligible effect of the microbiota on such dynamic events in the mouth or that the change in aromatic molecule release is not preceded by a shift in taxonomic composition. However, to appropriately test this hypothesis we would need longitudinal analysis of the interactions.

The molecular and biochemical changes in saliva related to obesity have not yet been comprehensively investigated. It has been reported that obesity is associated with oxidative stress caused by the uncontrolled uptake of nutrients [36], [37]. Oxidative stress results from an unbalance between antioxidants and the excessive production of reactive oxygen species that are not completely detoxified by cellular antioxidants. The simultaneous higher TAC and the elevated catalase expression in the salivary samples under investigation support the induction of a systemic antioxidant response in the obese subjects. It has also been reported that catalase activity, responsible for the elimination of H_2_O_2_ and the reduction of hydroxyl radicals, was significantly higher in the serum of obese compared with non-obese patients [38].

A high inter-individual variability was observed for protein content of the O and N salivary samples. The mean value of O and N protein content was not significantly different (P>0.05), but some trends were detected and will be discussed below.

Although wine has a limited impact on obesity, in this study, it was chosen as a model to resemble the in-mouth interaction between saliva and a food matrix (food matrix here defined according to the CE low N. 178/2002). This was to investigate the hypothesis that in obese individuals saliva could be responsible for an alteration of the retronasal aroma volatilization. There were several reasons for this choice: wine is a real food matrix; it is liquid and avoids food destructuring issues; it is fat free and not rich in proteins that would render the data interpretation more difficult; it is interestingly complex for the chemical variety of aroma compounds and glycoconjugated aroma precursors that contains. However, we recognize that choosing a liquid matrix we left out variables specifically related to the consumption of solid foods.

Our results showed that the volatility of the different chemical classes of wine odorants were suppressed after interaction with obese compared to normal-weight saliva. Esters, acetates and alcohols represent a major and common part of most fermentation compounds and their balance plays a significant role in wine aroma quality. Esters and particularly ethyl esters, are responsible for fruity odors, and their sensorial impact on wine characteristics is very important [39], [40] not only due to their single contribution, but also because they act synergistically when present together in mixtures [41]. Therefore, although our evidence comes from an *in vitro* study, the decrease in esters concentration suggests that perception intensity and quality of retronasal fruity aroma of white wine could be suppressed in obese people. Obviously, *in vivo* studies will be needed to validate this hypothesis. Among the identified alcohols, all involved together in the base aroma character of wines [42], [43], data concerning ethanol seems particularly interesting. The fact that its concentration is half of the average value detected for the group of normal-weight (P<0.05), suggests that obese people may perceive less alcohol content of wine and this, together with other psychological and physiological factors, might be a co-factor affecting the alcohol intake in the case of obesity [44]. Furthermore, also the small decreases observed for norisoprenoids may have a sensory impact. In fact, norisoprenoids are characterized by very low odor perception thresholds (vitispirane = 800 µg/L; TDN = 20 µg/L; β-damascenone = 0.05 µg/L) [45], [46] and it was observed, by reconstitution and omission tests, that fruity aroma notes in red wines can be enhanced by the presence of C13-norisoprenoids [47]. Therefore, the observed global decrease of norisoprenoids in obese could correspond to a further possible suppression in the perception of fruity notes, already strongly attenuated by the negative impact of their saliva on esters volatilization. Referring to aldehydes, furfural is a wine volatile linked to oxidation. Its significantly lower (P<0.05) mean concentration in the wine headspace after interaction with O compared to N saliva samples, could be linked to the higher TAC detected in saliva from obese subjects. A previous study [5] hypothesized that the basal concentrations of TAC in unstimulated saliva could affect the perception of certain substances; for example, those that are sensitive to oxidation. Our result on furfural seems to support this hypothesis.

All these evidences suggest that in obese subjects the olfactory perception in the mouth could be depressed/modified by the action of their saliva.

A PLS-DA was computed in order to find a model that separates classes of observations (O and N) on their X-variables (volatiles and biochemical parameters). A significant four-factor model was obtained and it shows that samples belonging to the two groups are clearly separated (Figure 5A and B; F3 and F4 not shown). In the score plot (Figure 5B), almost all the O samples are close to each other to form a single group; differently, the N samples, although grouped into two main areas, show a greater dispersion. This higher variability observed for N samples appears in agreement with the widely reported inter-individual variations of *in vivo* aroma release kinetics [14] and likely linked to the high degree of inter-individual variability in human saliva composition [5]. In contrast, the common trend observed in this study for O samples can be linked to a common alteration of the saliva composition occurring in cases of obesity. The loading plot (Figure 5A) shows that some biochemical variables (mainly TAC and total proteins) have higher levels in the saliva samples taken from O individuals. Moreover, based on the BW coefficients (Table 4), also albumin, transglutaminase E and lactotransferrin, together with the 2-phenylethyl acetate, are variables explaining O differences. The increase in these saliva proteins detected in most of O samples seems hence involved in the general reduction of in mouth volatiles release. It is well documented that proteins interact with volatile compounds [48]. The volatile-protein interactions occur in a variety of ways (ionic and hydrogen non-covalent interactions and steric trapping into hydrophobic areas) due to the considerable variation in the proteins amino acid side chain structures and tridimensional structure. Saliva proteins can interact with aroma compounds and consequently influence their partitioning between the liquid and air phase [49]. To the best of our knowledge, no information about the interaction of volatiles with lactotransferrin is available. On the contrary, due to its presence in several food matrices, it is known that the strength of hydrophobic interactions with albumin increases in line with the hydrophobicity of the aroma molecule [50]. Therefore, the higher concentration of albumin and of the total proteins, can explain the decreased in mouth volatile release after wine interaction with saliva from obese subjects. In addition, it is reported that lactotransferrin is endowed with anti-inflammatory and antioxidant activities, regulating parameters of oxidative stress and fat-induced inflammation in severely obese subjects [51]. Therefore, the high positive BW of lactotransferrin as well as TAC, suggest the induction of a systemic antioxidant response detectable in the saliva of obese subjects. As sated above, this antioxidant response could affect the perception of certain substances sensitive to oxidation. A further variable showing a high positive BW for O, is transglutaminase E. Transglutaminase is an enzyme naturally present in the majority of animal tissues and body fluids, and is involved in various biological processes. It acts on proteins by catalyzing reactions in the formation of covalent bonds between a carboxylamide group of the lateral chain on a Glutamine residue and an amino group of the lateral chain of a Lysine. These bonds may be formed between proteins of distinct types and origin. Thanks to these cross-linking capabilities, this enzyme is largely employed in the food industry (meat, fish and dairy products) in order to improve and standardize rheological properties and texture characteristics [52]. In the frame of this study, the higher amount of transglutaminase in the O saliva samples could enhance the “trapping” of volatiles by proteins as a consequence of the cross-linking process.

Saliva samples from N subjects were associated with higher amounts of different proteins (mainly cystatin-S and –SN, and cathepsin G) and generally, of volatiles (Figure 5A). Most of these volatiles (benzaldehyde, benzyl alcohol, ethyl acetate, hexanoic acid, 2-phenyethanol, furfural) resulted as variables with high positive BW indicating a significant better in-mouth volatilization of these wine aroma compounds after *in-vitro* interaction with N compared to O saliva. Also the high positive BW coefficient associated to alpha-amylase in N samples appears interesting, due to the possible sensory and nutritional implications previously reported. It seems that the amount of salivary alpha-amylase (an endo-enzyme making digestion of dietary starch in humans by hydrolyzing starch into maltose) affects both oral texture and flavor perception of starch foods. The individual differences in salivary amylase levels and salivary activity may contribute significantly to individual differences in dietary starch intake and, consequently, to overall nutritional status [53], [54].

The overall results obtained *in vitro* in this study with wine, suggest the possibility that the extent of the retronasal aroma release is suppressed in obese compared to normal weight subjects due to differences in saliva composition. Chemical interactions between the components of saliva and aroma molecules from white wine can reduce the in-mouth aroma volatilization in obese. It stands to reason that a significant reduction in the release of volatile chemical compounds from a food matrix such as wine would significantly alter the perception of that food matrix through the retronasal aroma sensation pathway. This study provides evidence that the saliva of people who have been clinically designated as obese (by BMI index and medical diagnosis) may reduce/modify their sensitivity to food aroma by reducing the release of volatile chemicals. However, this hypothesis remain to be tested on several food/drink matrices and in full clinical trials.

## Materials and Methods

### Sampling

Saliva was collected from 28 obese (O) and 28 normal weight (N) male individuals aged between 20 and 68 years (Table 1). Clinically designated obese individuals were recruited from the San Giovanni Bosco Hospital (ASL NA1, Naples, Italy), among bariatric surgery candidates. Normal weight participants were recruited among students, researchers and professors from the University of Naples Federico II. During a preparative consultation visit, candidates were given a flyer describing the study. Interested people completed a questionnaire asking them about diseases, medications, diet, allergy, and smoking habits and finally, their weight and their height were measured. Individuals with chronic diseases, allergies and under specific diets were excluded. Exclusionary criteria included any physiological condition or taking medications that could influence salivation (for example antidepressants and antihistamines). Also underweight (BMI<18.5) and overweight/pre-obese (25≥BMI≤29.9) candidates were excluded. All subjects gave written informed consent to participate after receiving oral and written information and the study protocol was approved by the Ethics Committee of Federico II University of Naples.

Participants were instructed not to consume food and beverages for at least 2.5 h prior to 10.30 a.m. Water drinking was allowed before the saliva collection started. Then, subjects were asked to wash teeth (new toothbrush and toothpaste were provided and were the same for all donors) and finally to rinse mouth several times with tap water to avoid any contamination from food/beverage residues and/or toothpaste flavorings. After 1.5 h from teeth brushing (12.00 a.m.), the collection of unstimulated whole saliva was started: subjects allowed their saliva to flow into sterile falcon tubes (50 mL). After collection of 5 mL of resting saliva (maximum collection time fixed at 30 min), the collection tube was tightly closed. After stirring, the tube was reopened and with the help of a sterile syringe, the saliva was divided into 3 aliquots for chemical, biochemical and microbiological investigations. Immediately after, the samples were stored at −20°C until the subsequent analyses. The collection was repeated twice and the samples were pooled per participant prior to further analyses.

### Microbiota analysis by 16 rRNA gene sequencing

Microbial DNA extraction was carried out by the BiosticTM Bacteremia DNA isolation kit (MoBIO Laboratories, Inc. Carlsbad, CA) using 1 mL of saliva. The V4 region of the 16S rRNA gene (515F-806R) was amplified using the Earth Microbiome Project barcoded primer set, adapted for the Illumina HiSeq2000 by paired-end sequencing and PCR conditions recently described [55]. The PCR amplifications were done in triplicate, and then pooled. Following pooling, amplicons were quantified using PicoGreen (Invitrogen) and a plate reader. Once quantified, different volumes of each of the products were pooled into a single tube so that each amplicon was represented equally. The pool was then cleaned using UltraClean PCR Clean-Up Kit (MoBIO), and then quantified using the Qubit (Invitrogen). After quantification, the molarity of the pool was determined and diluted to 2 nM, denatured, and then diluted to a final concentration of 4 pM with a 30% PhiX spike for loading on the Illumina HiSeq2000 sequencer.

### Bioinformatic analysis

Sequencing reads were demultiplexed according to perfect matches to the 12 bp index read encoding the barcode sequence. After discarding reads containing any ambiguous base calls or call with a confidence values of less than 99% (Phred score of Q20), 1,554,194 sequencing reads remained. Unless otherwise noted below, analyses were performed with scripts from the QIIME version 1.5.0 software suite [56] using default parameters. Operational taxonomic units (OTUs) clusters (97% identity) were identified using pick_otus.py, and then filtered for OTUs containing at least two sequences. The pick_rep_set.py and assign_taxonomy.py scripts were used to taxonomically classify each OTU based on alignment to the 4feb2011 release of the greengenes database. Assembly and interrogation of the OTU table using make_otu_table.py and per_library_stats.py revealed that the number of reads per sample varied from 35,283 to 4,256. Therefore all samples were rarified to 4,000 sequences per sample using single_rarefaction.py. Following construction of the OTUs into a phylogentic tree using align_seqs.py, filter_alignment.py, and make_phylogeny.py, summary graphs and visualizations were generated with beta_diversity_through_plots.py, summarize_taxa_through_plots.py and alpha_rarefaction.py as described by the online QIIME tutorial (http://qiime.org/tutorials/tutorial.html). Statistical tests for calculating the influence of sample variables on microbial ecology were applied using otu_category_significance.py (ANOVA) and compare_categories.py (ADONIS, ANOSIM, and MRPP). False discovery rate (FDR) was considered to evaluate the significance. Weighted UniFrac distance matrices were used for Principal Coordinate Analysis (PCoA) analysis. The OTU taxonomy table and the weighted UniFrac distance matrix generated by QIIME were used to produce heat maps by using the software TMeV v 4.8 [57].

### Biochemical analyses

The protein concentration was determined using Bradford protein assay according to manufacturer's instructions (Biorad, Milan, Italy).

The Total Antioxidant Capacity (TAC) of saliva samples was evaluated by Oxygen Radical Absorbance Capacity (ORAC) assay performed in 96-well microplates according to Gillespie et al. (2007) [58]. The assay was performed using Trolox (a water-soluble analogue of vitamin E) as standard at different concentrations (i.e. 6.25, 12.5, 25 and 50 µM). Samples were diluted 1∶100 in ice-cold 50% acetone and analyzed in quadruplicate. The fluorescence kinetic was measured at 37°C for 1 h, reading each minute, using a Synergy HT (Biotek,Winooski, USA) multi-detection reader at an excitation and an emission wavelength of 485 nm and 528 nm, respectively. The ORAC values are expressed as µM of Trolox Equivalents (TE) [58].

For Western blot analysis, two pooled samples were obtained by combining equal amounts (100 µL) of normal-weight and obese salivary samples. Proteins (15 µg) were resolved by 12% SDS-PAGE under reducing conditions and transferred onto nitrocellulose membrane (Sartorius, Göttingen, Germany) with an electroblot apparatus (Bio-Rad, Milan, Italy) as previously reported [59].. The following dilutions were used: anti-Catalase, 1∶4000 (catalog No. 041M4757); anti-SOD1, 1∶500 (catalog No. 1406464); anti-SOD2, 1∶500 (catalog No. 1406465). Immunoreactive protein bands were visualized by the enhanced chemiluminescence (ECL) Plus Western Blotting Detection System (GE Healthcare, Buckinghamshire, UK) according to the manufacturer's instructions.

For protein profiles analysis, samples were separated by analytical (15 µg) and preparative (200 µg) 12% SDS-PAGE electrophoresis [60]. Densitometric analyses were performed following gel acquisition on a ChemiDoc XRS station (Biorad). Molecular weight and intensity of each protein band were calculated on the normalized gels by using the ImageLab software (Biorad).

Selected protein bands showing the major variability were identified by Peptide Mass Fingerprinting (PMF) by MALDI-TOF Mass Spectrometry (MS) as previously reported [59], [61]. The protein identification was performed by using the Mascot software (version 2.2, Matrix Science, www.matrixscience.com). The following searching parameters were used: trypsin specificity for protein cleavage; mass tolerance 50 ppm; allowed number of missed cleavage sites up to 1; cysteine residues modified as carbamidomethyl-cys.

### Release of aroma compounds in the model mouth system

The in-mouth dynamic conditions were simulated by using a Retronasal Aroma Release (RAS) device equipped with an SPME fiber (Solid Phase Micro Extraction; Supelco Co., Bellefonte, USA), as previously described [6]. 30 mL of Falanghina white wine (pH: 3.19; total acidity: 6.15 g/L tartaric acid; residual sugars: 11.44 g/L; 13% v/v ethanol; vintage: 2007; winery: Cantine del Taburno, BN – Italy) together with 6 mL of whole saliva from obese or normal weight and 200 µL of an alcoholic solution of 2-octanol (50 mg into 250 mL of ethanol), employed as internal standard, were transferred into the sample flask (100 mL) of the model mouth system, which was kept in a water bath at 37°C. The wine sample without saliva was analyzed in order to identify volatiles. In this case, little glass balls were added in the RAS flask in order to maintain the headspace volume unchanged. In order to compare volatiles directly coming from saliva, O and N saliva samples (mix of all donors belonging to each of the two categories) without wine were analyzed. During this last analysis, 30 mL of mQ water were added to the 6 mL of saliva in order to consider volume and dilution conditions. The SPME fiber was inserted into the sample container through a septum and then exposed to the headspace. A purified nitrogen flow (20 mL/s) passed through the wine/saliva mixture for 10 min, while volatile compounds were trapped on a conditioned (250°C for 3 h in a GC injection port) SPME fiber (DVB/CAR/PDMS; 50/30 µm thickness; coating phase; 2 cm length). The absence of extraneous/residual molecules on the fiber was checked before each analysis. We conducted the saliva/wine interaction experiment per subject and we replicated twice.

### High resolution gas chromatography analyses (HRGC/MS and HRGC/FID)

Volatiles adsorbed on the SPME fiber's coating phase were desorbed in split-splitless mode (split valve opened at 11 min and closed at 25 min) at 250°C for 10 min in the injection port of a GC/MS-QP2010 quadrupole mass spectrometer (Shimadzu, Shimadzu corp., Kyoto, Japan) equipped with a DB-WAX column (60 m×0.250 i.d., 0.25 µm film thickness; J&W Scientific Inc., Folsom, CA 95360, USA). The temperature program used was 40°C for 5 min, raised at 2°C/min to 220°C, and held for 20 min at maximum temperature, starting immediately after exposure of the SPME fiber in the RAS device. The injector port and the ion source were maintained at 250 an 230°C, respectively. The carrier gas used was helium (1.3 mL/min). Electron impact mass spectra were recorded with ion source energy of 70 eV and peak areas were measured using a GC/MS solution program Shimadzu version 2.30 (Shimadzu corp., Kyoto, Japan). Compounds identification and concentration measurement were performed as previously reported [6]. Quantitative analysis of volatiles was performed under the same chromatographic conditions by a 7890A GC-System equipped with a FID detector (Agilent Technologies, Palo Alto, USA).

### Data analysis

Significant differences between the two study groups (O vs. N) were determined within microbiological, biochemical and chemical parameters, by means of the non-parametric Wilcoxon-Mann-Whitney test for populations whose data cannot be assumed to be normally distributed [62], [63].

Partial least square discriminate analysis (PLS-DA) was chosen as an exploratory technique to investigate the separation between groups of observations and to understand which variables contains the information allowing the separation. The PLS-DA was computed to get an indication on which volatiles and salivary biochemical parameters associated best with the O or N class. Therefore, the multivariate calibration modeling was performed with quantitative data concerning significantly different volatiles (as reported in Table 3), differently expressed proteins, total proteins and TAC as predictive variables (X), across the O and N saliva samples as dependent categorical response variables (Y). The matrix Y contained the values 1 and 0 as dummy variables for the O and N individuals, respectively. As a data pretreatment, normalization using the 1/SDEV transform to treat all parameters as having equal potential influence was used. A cross-validation procedure to determine the maximum number of significant dimensions was applied. Martens' uncertainty test option was considered to check for variables' significance. High-weighted regression coefficients or BW were used to identify significant parameters predicted by the model [64].

Statistical data processing was performed using the Unscrambler®, v. 10.2, CAMO Software A/S, (Trondheim, Norway). Throughout all data analyses, differences were considered to be significant at a level P<0.05.
